# Palliative Systemic Therapy Given near the End of Life for Metastatic Non-Small Cell Lung Cancer

**DOI:** 10.3390/curroncol29030112

**Published:** 2022-02-23

**Authors:** Marc-Étienne Beaudet, Yves Lacasse, Catherine Labbé

**Affiliations:** Institut Universitaire de Cardiologie et de Pneumologie de Québec, Université Laval, Québec, QC G1V 4G5, Canada; marc-etienne.beaudet.1@ulaval.ca (M.-É.B.); yves.lacasse@fmed.ulaval.ca (Y.L.)

**Keywords:** palliative systemic therapy, end of life, non-small cell lung cancer

## Abstract

Background: The use of chemotherapy near end of life (EOL) for various cancers is increasing and has been shown to be associated with delayed access to palliative care (PC) and increased aggressiveness in EOL care, without any benefit on survival. Methods: This retrospective study included 90 patients with metastatic non-small cell lung cancer (NSCLC) who received at least one line of palliative systemic anticancer therapy (SACT) and died between 1 November 2014, and 31 October 2016, at Institut universitaire de cardiologie et de pneumologie de Québec (IUCPQ). Our primary objective was to evaluate the proportion of patients with NSCLC receiving SACT within 30 days of death. Secondary outcomes were to determine the mean and median delays between the administration of the last treatment and death, and to evaluate if there were differences in characteristics and outcomes (including overall survival (OS)) between patients treated or not within 30 days of death. Results: In our cohort, 22% of patients received SACT within 30 days of death. For the entire cohort, the mean delay between the last treatment and death was 94 days, and the median was 57 days. There were no statistically significant differences between the two groups in terms of baseline characteristics. Use of SACT near EOL was associated with decreased access to PC, higher rates of in hospital death, decreased use of medical aid in dying (MAiD), and a shorter median OS (4.0 vs. 9.0 months). Conclusions: In this retrospective cohort of patients with metastatic NSCLC, 22% of patients received SACT within 30 days of death, with a negative impact on access to PC, higher rates of in hospital death, decreased use of MAiD and palliative sedation, and a shorter median OS.

## 1. Introduction

Lung cancer is the most frequently diagnosed cancer and the leading cause of cancer death worldwide [1], with a 5-year survival of 19% [2]. Non-small cell lung cancer (NSCLC) is the predominant form of the disease, accounting for approximately 85% of cases [3]. Among newly diagnosed patients with NSCLC, 40% present with metastatic disease [4]. The ultimate objective of treating advanced NSCLC with the sequential use of systemic anticancer therapies (SACTs) is to improve overall survival (OS) while maintaining or improving quality of life.

In the last decade, targeted therapies with tyrosine kinase inhibitors (TKIs) have become standard first-line therapy for patients with driver oncogenes, with median survival in phase 3 trials beyond 3 years for patients with epidermal growth factor receptor (*EGFR*) mutations [5,6], and beyond 4 years for those with anaplastic lymphoma kinase (*ALK*) rearrangements [7,8]. Another significant advance was the use of immunotherapy to target immune checkpoint pathways to prevent or reduce tumor-mediated immune suppression. Nivolumab, pembrolizumab (for patients with programmed cell death ligand (PD-L1) ≥ 1%) and atezolizumab were all initially approved in 2015 and 2016 for second-line therapy of NSCLC after phase 3 trials showing their superiority over docetaxel [9,10,11,12]. Recently, immunotherapy became a standard of care for all patients in the first-line setting unless contraindicated, as a monotherapy or in combination with chemotherapy, depending on PD-L1 expression [13].

Even if these treatments might prolong survival or reduce symptoms, not all patients will benefit, and many will experience adverse effects. Furthermore, SACT might prevent the patient from preparing for death, delay access to palliative care (PC) or preclude entry into hospice [14,15]. Multiple studies show that palliative chemotherapy is increasingly given near death for incurable cancer, with a non-negligible impact on healthcare costs [16]. In a US community practice in 2006, chemotherapy for advanced NSCLC was given within 1 month and 2 weeks of death to 43% and 20% of patients, respectively [17]. There is also literature showing that patients receiving targeted therapy for metastatic NSCLC within 30 days of death are more likely to undergo aggressive end-of-life (EOL) care, including multiple emergency visits, prolonged hospitalization, admission to intensive care units, and late hospice referrals [18,19]. Similar data exist for immune checkpoint inhibitors, but not specifically for patients with NSCLC. Their use in the last 30 days in 157 patients with multiple tumor sites was associated with poor Eastern Cooperative Oncology Group performance status (ECOG PS), lower hospice enrollment, and dying in the hospital [20]. The rate of chemotherapy administration near the EOL has been proposed as an indicator for the assessment of quality of care in cancer patients [21], but these recommendations were made before the widespread use of TKIs and immunotherapy.

Given the lack of recent literature on the subject, we sought to explore the use and impacts of SACT including chemotherapy, TKIs and immunotherapy near EOL, specifically in patients with NSCLC. Our primary objective was to evaluate the proportion of patients with NSCLC receiving palliative SACT within 30 days of death at our center. Secondary outcomes were to determine the mean and median delays between the administration of the last treatment and death, and to evaluate if there were differences in characteristics and outcomes (including OS) between patients treated or not within 30 days of death.

## 2. Materials and Methods

### 2.1. Study Design

This retrospective study included all patients with metastatic NSCLC who received at least one line of palliative SACT and who died between 1 November 2014, and 31 October 2016, at Institut universitaire de cardiologie et de pneumologie de Québec (IUCPQ). Patients were identified from the Oncology Database (SICTO), which is a regional database in Quebec City, and data collection was performed from chart review. The study was approved by our institutional Research Ethics Committee.

### 2.2. Data Collection

Demographic data collected for this study included age, gender, and smoking status. The medical charts were also reviewed for histology, biomarker results (*EGFR*, *ALK*, PD-L1), stage at initial diagnosis, number of organs involved, presence of brain metastasis. Information on treatment and outcomes were collected: use of palliative radiation, type and number of lines of systemic therapy received, ECOG PS and level of care at last cycle before death, reason for treatment discontinuation, PC team involvement, as well as cause, place and date of death.

### 2.3. Statistical Analysis

Patient demographics and clinical characteristics were summarized using descriptive methods. Data were expressed using mean ± standard deviation (SD) or median ± interquartile range (IQR) for continuous variables, or as percentage for categorical data. Throughout the analysis, we compared patients who received palliative SACT within 30 days of death to those who did not. Categorical and continuous variables were compared using Fisher’s exact test and one-way analysis of variance, respectively. We constructed survival curves using Kaplan-Meier estimates, and used the log-rank test for between-group comparisons. Cox proportional hazard regression analysis was performed to model survival at follow-up, with adjustment for baseline characteristics and comorbidities. Variables with a probability value < 0.20 were candidates for multivariable regression modelling using a forward approach. We tested the assumption of proportional hazards using cumulative sums of Martingale residual plots. Statistical significance was present with a two-tailed *p* value < 0.05. Analyses were performed using SAS version 9.4 (SAS Institute Inc., Cary, NC, USA).

## 3. Results

### 3.1. Patients

**Figure 1:**
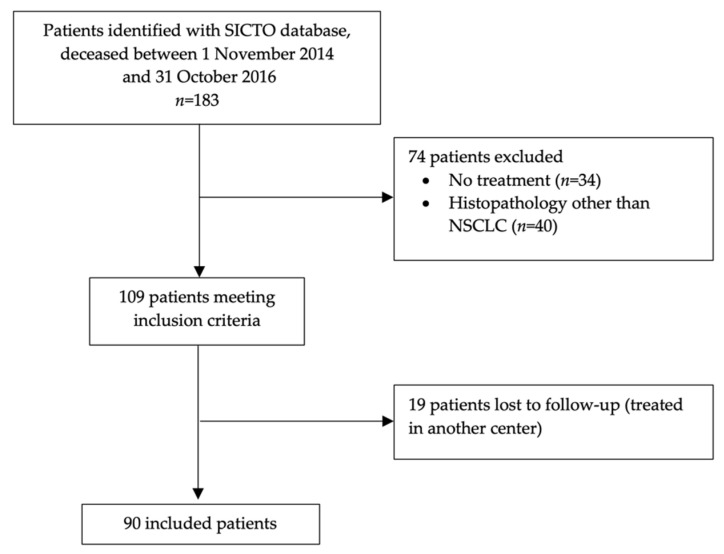
Patient-flow diagram.

One hundred and 83 patients were identified from the Oncology Database. After excluding 34 patients who did not receive SACT and 40 patients with a histopathological diagnosis other than NSCLC, 109 patients were eligible. Of these, 19 patients were diagnosed at our center but were transferred to their referring institution for treatment and lost to follow-up. Hence, 90 patients were included in the analysis (Figure 1).

Baseline characteristics of all patients are summarized in Table 1. Most patients were male, former or current smokers, and had an adenocarcinoma without a driver alteration. Only 12% of patients carried an *EGFR* mutation and 3% an *ALK* rearrangement. PD-L1 status was unknown for 85% of patients, as at the time first-line immunotherapy was not available, and second-line nivolumab was approved for all comers. The population was divided in two groups, with 20 patients (22%) who received SACT within 30 days of death, and 70 (78%) who did not. There were no statistically significant differences between the two groups. There were a higher proportion of adenocarcinoma (85% vs. 71%) and *ALK* rearrangements (10% vs. 1%) in the group receiving treatment near EOL, but the differences were not significant (both *p* values = 0.05).

### 3.2. Systemic Treatments

Lines and duration of systemic therapy are shown in Table 2. The most frequently use regimens were platinum-doublet chemotherapy in first line (81%) and immunotherapy in second line (58%). Median number of cycles received and duration of treatment decreased with increasing lines of treatment.

### 3.3. Treatments and Outcomes According to the Timing of Last Systemic Therapy

For the entire cohort, the mean delay between the last treatment and death was 94 days, and the median was 57 days. When compared with patients not treated near EOL, patients receiving SACT within 30 days of death received similar numbers of lines of therapy, and a similar proportion of immunotherapy (Table 3). There was a tendency for an increased use of TKIs in the group treated near EOL, which was not statistically significant. There were no significant differences between the two groups in terms of ECOG PS and level of care. Patients not treated in the last 30 days of life were more likely to be seen by the PC team, and to receive medical aid in dying (MAiD) or palliative sedation. They also were more likely to die in a hospice or at home, as opposed to at the hospital. Only one patient died from treatment toxicity, in the group treated near EOL.

**Figure 2:**
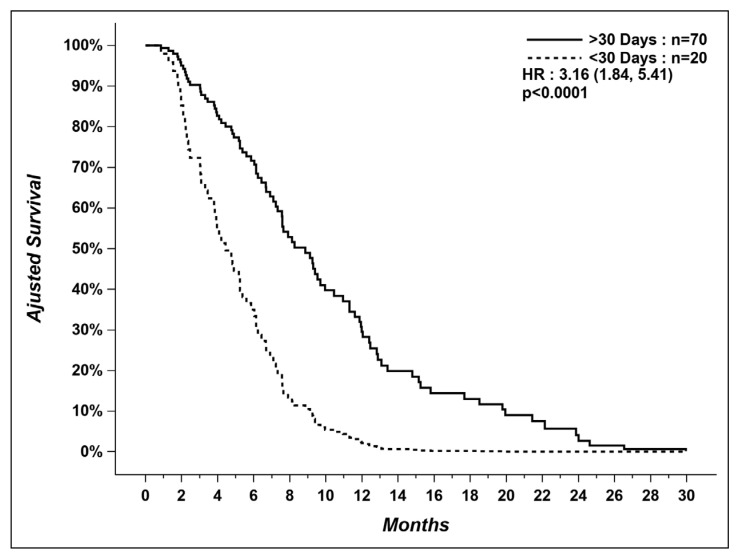
Overall survival of patients who received systemic therapy within last 30 days versus patients who did not.

After adjusting for other factors influencing OS in our multivariable model, patients not treated in the last 30 days of life had a longer median OS (time from diagnosis of metastatic disease to death) than patients receiving SACT within 30 days of death (9.0 vs. 4.0 months) (Figure 2).

**Figure 3:**
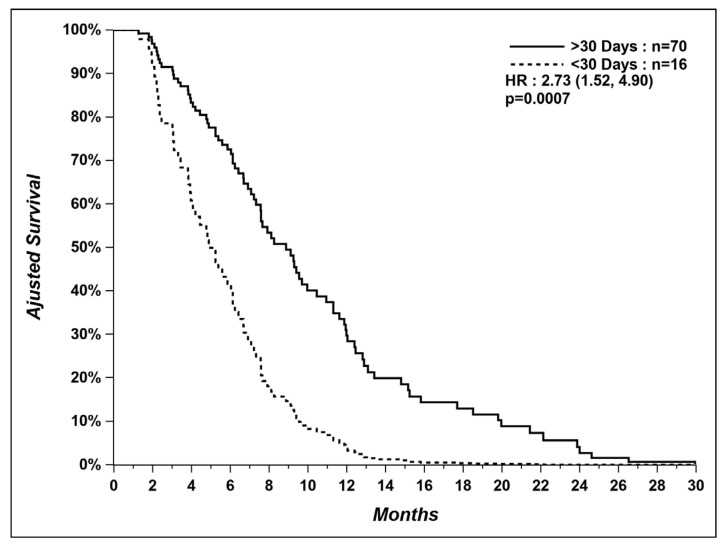
Overall survival of patients who received systemic therapy within last 30 days versus patients who did not, after excluding patients with unexpected and unknown causes of death.

In the group of patients treated in the last 30 days, we could not find the cause of death for two patients, and two other patients died from unexpected causes (1 arrived at the emergency room with cardiopulmonary arrest and 1 died from pulmonary hemorrhage caused by anticoagulation for atrial fibrillation). Even after excluding these four patients, patients not treated in the last 30 days still had a longer median OS (9.0 vs. 4.4 months) (Figure 3).

## 4. Discussion

In this retrospective study of patients receiving SACT for metastatic NSCLC, 22% of patients were treated within 30 days of death. This study being retrospective, it was not easy to understand why these patients were treated near EOL, as there were no statistically significant differences in terms of baseline characteristics between these patients and those not treated within 30 days of death. However, there was a tendency for a higher proportion of patients with *EGFR* mutations and *ALK* rearrangements and for an increased use of TKIs in the group treated near EOL. It is not uncommon to continue TKI therapy beyond progression in the clinical setting, and this approach has shown survival benefits compared with switching to chemotherapy [22,23]. Patients can still experience quick clinical deterioration when using this strategy, and this might be an explanation for some of the rapid deaths in the group treated near EOL. There was also a tendency for a higher proportion of patients on first-line therapy and a lower proportion on third line or beyond in the group treated within 30 days of death. This could be explained by a higher proportion of patients with more aggressive disease leading to rapid and/or unexpected deterioration. There is consistent evidence that a significant proportion of patients with non-oncogene-addicted advanced NSCLC derive no or only limited benefit from first-line chemotherapy [24].

Similar to what has been reported in other studies [14,15,18,19,20], the use of SACT near EOL had a negative impact on PC access, which is unfortunate with the growing evidence for the benefits of PC in patients with advanced cancer. Temel and colleagues [25] published a landmark randomized clinical trial showing that patients with NSCLC who received PC referral at time of diagnosis had improved quality of life, mood, and increased survival, with less use of aggressive medical treatments at the end of life. The American Society of Clinical Oncology (ASCO) recommend that all patients with advanced cancer receive integrated PC services early in their disease course [26]. Unfortunately, a Canadian study showed that PC is not accessed early or systematically in Canada [27], as confirmed in our small cohort.

In our study, there was also a tendency for a higher proportion of patients dying in the hospital when receiving SACT within last 30 days of life, which has also been proposed as an indicator for the assessment of quality of care in cancer patients [21]. There is a paucity of recent data regarding the place of death in patients with lung cancer and other tumor types, but the proportion of patients dying in an acute hospital ranges from 28% to 60% [28,29,30]. Our numbers seem higher than what has been reported, which might be explained by many factors. MAiD and palliative sedation are only performed in hospital in our city. Furthermore, our hospital has a palliative care ward which is similar to a hospice and most patients admitted on this ward are terminally ill. There were significant differences between our two groups of patients in the cause of death, with patients receiving MAiD or palliative sedation only in the group not having received SACT near EOL. MAiD and palliative sedation are part of EOL care that should be accessible for all patients.

Our study also showed that patients receiving SACT within 30 days of death had a significant shorter survival compared to patients who had their last treatment > 30 days before death (4.0 vs. 9.0 months). Other studies have found no benefit of SACT near EOL on survival [15,31], but to our knowledge, this is the first report suggesting a detrimental effect. It is hard to determine if the shorter survival observed is explained by SACT, or by decreased access to PC, or by other factors, such as quick clinical deterioration when continuing TKIs beyond progression or different tumor biology/aggressiveness, as discussed earlier.

### Limitations

Our results are limited by the retrospective and unicentric nature of the study. Furthermore, our sample was small, probably explaining lack of power to demonstrate significant differences between our two groups of patients. The proportion of patients with driver alterations was low, so we could not make any conclusions specifically on the use of targeted therapy near EOL. We had no information on patient-reported outcomes and quality of life. Additionally, the study was performed on patients treated between 2014 and 2016, before the widespread use of immunotherapy as a first-line treatment, as a monotherapy or in combination with chemotherapy.

## 5. Conclusions

In this retrospective cohort of patients with metastatic NSCLC who received at least one line of palliative SACT and died between 2014 and 2016, 22% were treated within 30 days of death. Receiving treatment near EOL was associated with decreased access to PC, higher rates of in hospital death, decreased use of MAiD and palliative sedation, and a shorter median OS.

With rapidly evolving treatment options and new algorithms for the treatment of metastatic NSCLC, more studies are needed to assess the use of various SACT near EOL and its impact on outcomes. Still, our results show that a significant proportion of patients with advanced NSCLC continue to receive SACT near death, and likely reflect recent patterns of EOL care for patient with lung cancer in Canada. There is a need for increased and early integration of palliative care for patients.

## Figures and Tables

**Table 1:** Baseline patient characteristics and outcomes.

Characteristic	All Patients (*n* = 90)	SACT Within Last 30 Days (*n* = 20)	No SACT Within Last 30 Days (*n* = 70)	*p* Value
Mean age, years, ±SD	67 ± 7	69 ± 6	66 ± 7	0.17
Male sex	46 (51%)	11 (55%)	35 (50%)	0.80
Former or current smoker	82 (91%)	17 (85%)	65 (93%)	0.52
Histology				0.05
Adenocarcinoma	67 (74%)	17 (85%)	50 (71%)	
Squamous cell carcinoma	14 (16%)	0	14 (20%)	
Other *	9 (10%)	3 (15%)	6 (9%)	
*EGFR* mutation				0.21
Positive	11 (12%)	3 (15%)	8 (11%)	
Negative	62 (69%)	16 (80%)	46 (66%)	
Not tested (squamous carcinoma)	14 (16%)	0	14 (20%)	
Unknown	3 (3%)	1 (5%)	2 (3%)	
*ALK* rearrangement				0.05
Positive	3 (3%)	2 (10%)	1 (1%)	
Negative	70 (78%)	17 (85%)	53 (76%)	
Not tested (squamous carcinoma)	14 (16%)	0	14 (20%)	
Unknown	3 (3%)	1 (5%)	2 (3%)	
PD-L1 status				0.72
<1%	4 (4%)	1 (5%)	3 (4%)	
1–49%	1 (1%)	0	1 (1%)	
≥50%	9 (10%)	3 (15%)	6 (9%)	
Unknown	76 (85%)	16 (80%)	60 (86%)	
Stage IV at diagnosis	77 (86%)	17 (85%)	60 (86%)	1
Number of organs involved, including lung				0.70
1	23 (26%)	4 (20%)	19 (27%)	
2	38 (42%)	10 (50%)	28 (40%)	
≥3	29 (32%)	6 (30%)	23 (33%)	
Brain metastasis	25 (28%)	3 (15%)	22 (31%)	0.33

*ALK* = anaplastic lymphoma kinase; *EGFR* = epidermal growth factor receptor; PD-L1 = programmed death ligand 1; SACT = systemic anticancer therapy; SD = standard deviation. * Poorly differentiated carcinoma (*n* = 6), adenosquamous carcinoma (*n* = 3).

**Table 2:** Lines and duration of systemic therapy.

	Lines of Therapy			
	1	2	3	>3
Patients, n (%)	90 (100)	39 (43)	12 (13)	5 * (6)
Therapy received				
Platinum-pemetrexed	54	3	1	1
Platinum-gemcitabine	19	0	0	0
Gefitinib	10	2	0	0
Erlotinib	1	0	0	0
Osimertinib	0	0	1	0
Crizotinib	2	1	0	0
Ceritinib	0	0	0	1
Pemetrexed	1	0	0	0
Gemcitabine	1	0	1	1
Nivolumab	1 **	19	2	0
Pembrolizumab	0	6	0	0
Docetaxel	1	8	3	0
Vinorelbine	0	0	4	2
Median number of cycles (IQR)	4	3	3	2
	(2–5)	(2–6)	(3–6)	(1–2)
Median duration of treatment, days (IQR)	63	54	63	34
	(27–105)	(21–84)	(37–104)	(18–34)

* Four patients received 4 lines of therapy, and 1 patient received 6 lines. ** Immediate progression after chemoradiation for stage III disease.

**Table 3:** Treatments and outcomes according to timing of last systemic therapy.

	All Patients (*n* = 90)	SACT Within Last 30 Days (*n* = 20)	No SACT Within Last 30 Days (*n* = 70)	*p* Value
Palliative radiation	35 (39%) *	4 (20%)	31 (44%)	0.20
Lung	6 (7%)	0	6 (9%)	
Brain	14 (16%)	2 (10%)	12 (17%)	
Bone	17 (19%)	3 (15%)	14 (20%)	
Line of therapy at time of death				0.89
1	51 (56%)	13 (65%)	38 (54%)	
2	27 (30%)	6 (30%)	21 (30%)	
3	7 (8%)	1 (5%)	6 (9%)	
>3	5 (6%)	0	5 (7%)	
Therapy received during course of metastatic disease **				
Chemotherapy only	50 (56%)	9 (45%)	41 (59%)	0.32
≥1 line of immunotherapy	28 (31%)	6 (30%)	22 (31%)	1.00
≥1 line of TKI	14 (16%)	5 (25%)	9 (13%)	0.29
Therapy received within last 30 days				
1st line doublet chemotherapy		5 (25%)		
1st line single agent chemotherapy		1 (5%)		
Maintenance chemotherapy		2 (10%)		
1st line TKI		5 (25%)		
2nd line immunotherapy		6 (30%)		
3rd line chemotherapy		1 (5%)		
ECOG PS at last cycle before death				0.81
1	53 (59%)	13 (65%)	40 (57%)	
2	28 (31%)	6 (30%)	22 (32%)	
Unknown	9 (10%)	1 (5%)	8 (11%)	
Level of care at last cycle before death ^§^				0.59
1	8 (9%)	1 (5%)	7 (10%)	
2	19 (21%)	6 (30%)	13 (19%)	
Not discussed	63 (70%)	13 (65%)	50 (71%)	
Reason for last treatment discontinuation				**<0.01**
Disease progression	43 (48%)	1 (5%)	42 (60%)	
Toxicity	21 (23%)	1 (5%)	20 (29%)	
Death	26 (29%)	18 (90%)	8 (11%)	
PC team involvement before death	80 (89%)	14 (70%)	66 (94%)	**0.01**
Cause of death				**0.0004**
Lung cancer	75 (84%)	15 (75%)	60 (86%)	
Toxicity	1 (1%)	1 (5%)	0	
MAiD or palliative sedation	10 (11%)	0	10 (14%)	
Other ^¶^	2 (2%)	2 (10%)	0	
Unknown	2 (2%)	2 (10%)	0	
Place of death				**0.0406**
Hospital	71 (79%)	17 (85%)	54 (77%)	
Hospice	14 (16%)	1 (5%)	13 (19%)	
Home	3 (3%)	0	3 (4%)	
Unknown	2 (2%)	2 (10%)	0	

ECOG PS = Eastern Cooperative Oncology Group performance status; MAiD = medical aid in dying; PC = palliative care; SACT = systemic anticancer therapy; TKI = tyrosine kinase inhibitor. * Two patients had palliative radiation to both brain and bone. ** Two patients received both immunotherapy and TKIs. ^§^ Level 1 is provision of maximal interventions offered by the treating team (including chest compressions, intubation and critical care unit transfer). Level 2 is provision of maximal interventions, with some restrictions (usually exclusion of chest compressions and/or intubation and/or critical care unit transfer). Level 3 is comfort care. ^¶^ One patient presented to the emergency room with cardiopulmonary arrest. One patient with atrial fibrillation on anticoagulation died from pulmonary hemorrhage.

## Data Availability

The data presented in this study are available on request from the corresponding author.
